# Supplementary material for: AMPK-mediated HCN4 channel phosphorylation contributes to age-related intrinsic bradycardia
Source: J Gen Physiol. 2026 Feb 6;158(2):e202513873. doi: 10.1085/jgp.202513873 (PMC12880560; doi:10.1085/jgp.202513873)
Supplement: Table S1 — shows data from If experiments in isolated SAN cells. [file jgp_202513873_tables1.pdf]

Fig	Var.	Condition	Shapiro-Wilk	Norm	Comp. test	Comparison	p	Significance
1	V1/2	Female ctrl	0.9921	Yes	LMM (normal)			
		Female + AICAR	0.3686	Yes		Female ctrl vs AICAR	0.2990	n.s.
		Male ctrl	0.3080	Yes		Male ctrl vs AICAR	0.6737	n.s.
		Male + AICAR	0.3520	Yes				
	Id	Female ctrl	0.0795	Yes	GLMM (Gamma)	Female ctrl vs AICAR	0.0982	n.s.
		Female + AICAR	0.4115	Yes		Male ctrl vs AICAR	0.0030	**
		Male ctrl	0.0264	No		Male ctrl vs Female ctrl	0.5067	n.s.
		Male + AICAR	0.0503	Yes				
7	V1/2	Young ctrl	0.5984	Yes	LMM (normal)			
		Young + AICAR	0.4127	Yes		Young ctrl vs Young + AICAR	0.0819	n.s.
		Old ctrl	0.3231	Yes		Old ctrl vs Old + AICAR	0.5354	n.s.
		Old + AICAR	0.3167	Yes				
	Id	Young ctrl	0.5150	Yes	GLMM (Gamma)	Young ctrl vs Young + AICAR	0.0037	**
		Young + AICAR	0.0023	No		Old ctrl vs Old + AICAR	0.9894	n.s.
		Old ctrl	0.0048	No		Young ctrl vs Old ctrl	0.0112	*
		Old + AICAR	0.0010	No				
8	V1/2	Young ctrl	0.5621	Yes	LMM (normal)			
		Young + Comp.C	0.2060	Yes		Young ctrl vs Young + Comp.C	0.9120	n.s.
		Old ctrl	0.6455	Yes		Old ctrl vs Old + Comp.C	0.4645	n.s.
		Old + Comp.C	0.9827	Yes				
	Id	Young ctrl	<0.0001	No	GLMM (Gamma)	Young ctrl vs Young + Comp.C	0.9986	n.s.
		Young + Comp.C	0.0058	No		Old ctrl vs Old + Comp.C	0.0110	*
		Old ctrl	<0.0001	No		Young ctrl vs Old Comp.C	0.9975	n.s.
		Old + Comp.C	0.0032	No				

Table S1. Data from I_f experiments in isolated SAN cells. These data were obtained from multiple mice and multiple cells per mouse, and we therefore included the mouse identifier as a random effect. Shapiro-Wilk normality test revealed both normal and non-normal distributions. Data were analyzed using a Linear Mixed Model (LMM) or Generalized Linear Multiple Model (GLMM), respectively. We used visual plots to assess the validity of the choice of the used model. For all analyses involving more than two pairwise comparisons, we reported adjusted p-values using Tukey's post hoc method. V1/2: half activation voltage; Id: I_f current density.
