# Supplementary material for: AMPK-mediated HCN4 channel phosphorylation contributes to age-related intrinsic bradycardia
Source: J Gen Physiol. 2026 Feb 6;158(2):e202513873. doi: 10.1085/jgp.202513873 (PMC12880560; doi:10.1085/jgp.202513873)
Supplement: Table S2 — shows data from IHCN4 experiments in HEK293 cells. [file jgp_202513873_tables2.pdf]

| Fig | Var. | Condition | Shapiro-Wilk | Norm |
|---|---|---|---|---|
| 2 | V1/2 | HEK T ctrl | 0.8466 | Yes |
| | | HEK T + AICAR | 0.0699 | Yes |
| | Id | HEK T ctrl | 0.0022 | No |
| | | HEK T + AICAR | 0.0012 | No |
| 3A | Id | ctrl | 0.0069 | No |
| | | AICAR | 0.4597 | Yes |
| | | Comp.C | 0.2342 | Yes |
| 3B | Id | ctrl | 0.5845 | Yes |
| | | AICAR | 0.0005 | No |
| | | AICAR + Comp.C | 0.1741 | Yes |
| 4A | Id | wt ctrl | 0.4756 | Yes |
| | | wt + AICAR | 0.0304 | No |
| | | S1157A ctrl | 0.5427 | Yes |
| | | S1157A + AICAR | 0.3999 | Yes |
| 4B | Id | wt ctrl | 0.8112 | Yes |
| | | wt + AICAR | 0.4509 | Yes |
| | | S1157D ctrl | 0.2912 | Yes |
| | | S1157D + AICAR | 0.6937 | Yes |
| 5A | Id | wt ctrl | 0.3362 | Yes |
| | | wt + AICAR | 0.0339 | No |
| | | S1158A ctrl | 0.6653 | Yes |
| | | S1158A + AICAR | 0.2982 | Yes |
| 5B | Id | wt ctrl | 0.4123 | Yes |
| | | wt + AICAR | 0.8207 | Yes |
| | | S1158D ctrl | 0.3103 | Yes |
| | | S1158D + AICAR | 0.4843 | Yes |
| 6 | Id | wt ctrl | 0.2499 | Yes |
| | | wt + AICAR | 0.5644 | Yes |
| | | Dbl mut ctrl | 0.1574 | Yes |
| | | Dbl mut + AICAR | 0.9243 | Yes |

| Comp. test | Comparison | p | Significance |
|---|---|---|---|
| LM (Normal) | ctrl vs AICAR | 0.6105 | n.s. |
| GLM (Gamma) | ctrl vs AICAR | <0.0001 | **** |
| GLM (Gamma) | ctrl vs AICAR | 0.0001 | **** |
| | ctrl vs Comp.C | 0.7688 | n.s. |
| | AICAR vs Comp.C | <0.0001 | **** |
| GLM (Gamma) | ctrl vs AICAR | 0.0243 | * |
| | ctrl vs Comp.C | 0.9961 | n.s. |
| | AICAR vs AICAR + Comp.C | 0.0149 | * |
| GLM (Gamma) | wt ctrl vs wt + AICAR | 0.0004 | *** |
| | wt ctrl vs S1157A ctrl | 1 | n.s. |
| | S1157A ctrl vs S1157A + AICAR | 0.9974 | n.s. |
| LM (Normal) | wt ctrl vs wt + AICAR | 0.0263 | * |
| | wt ctrl vs S1157D ctrl | 0.8840 | n.s. |
| | S1157D ctrl vs S1157D + AICAR | 0.9219 | n.s. |
| GLM (Gamma) | wt ctrl vs wt + AICAR | 0.0065 | ** |
| | wt ctrl vs S1158A ctrl | 0.6143 | n.s. |
| | S1158A ctrl vs S1158A + AICAR | 0.0136 | * |
| LM (Normal) | wt ctrl vs wt + AICAR | <0.0001 | **** |
| | wt ctrl vs S1158D ctrl | 0.9340 | n.s. |
| | S1158D ctrl vs S1158D + AICAR | 0.0007 | *** |
| LM (Normal) | wt ctrl vs wt + AICAR | 0.0037 | ** |
| | wt ctrl vs Dbl mut ctrl | 0.0020 | ** |
| | Dbl mut ctrl vs Dbl mut+AICAR | 0.9365 | n.s. |

**Table S2. Data from $I_{HCN4}$ experiments in HEK293 cells.** Shapiro-Wilk normality test revealed both normal and non-normal distributions for $I_{HCN4}$ current density (Id). Half activation voltage (V1/2) distributions in Figure 2 were normal and were compared with a t-test analysis. Only current density (Id) distributions were analyzed in Figures 3 through 6. Id data were analyzed using either a Generalized Linear Model (GLM-Gamma) when at least one of the distributions was not normal, or a Linear Model (Normal) if all distributions were normal. We used visual plots to assess the validity of the choice of the used model. When testing differences between three or more groups, as for Id data in Figures 3 through 6, we adjusted the between-group comparisons using Tukey's post hoc multiple comparisons.
