# Supplementary figures and images for: AMPK-mediated HCN4 channel phosphorylation contributes to age-related intrinsic bradycardia
Source: J Gen Physiol. 2026 Feb 6;158(2):e202513873. doi: 10.1085/jgp.202513873 (PMC12880560; doi:10.1085/jgp.202513873)

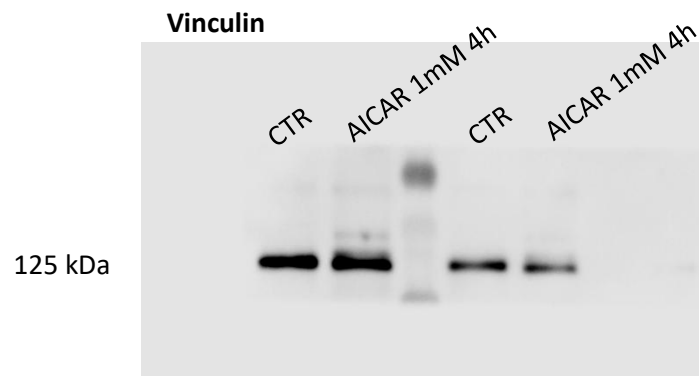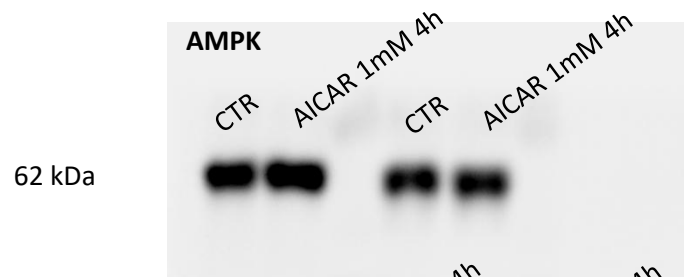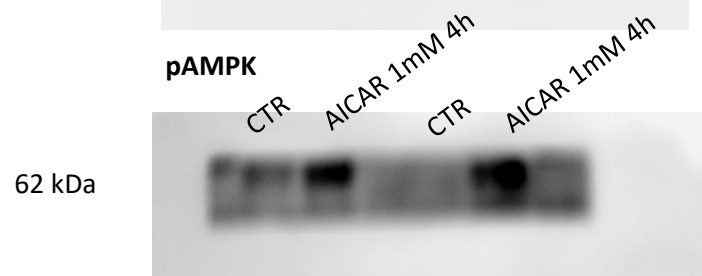

Supplement: SourceData FS1 — is the source file for Fig. S1. [file jgp_202513873_sourcedatafs1.pdf]

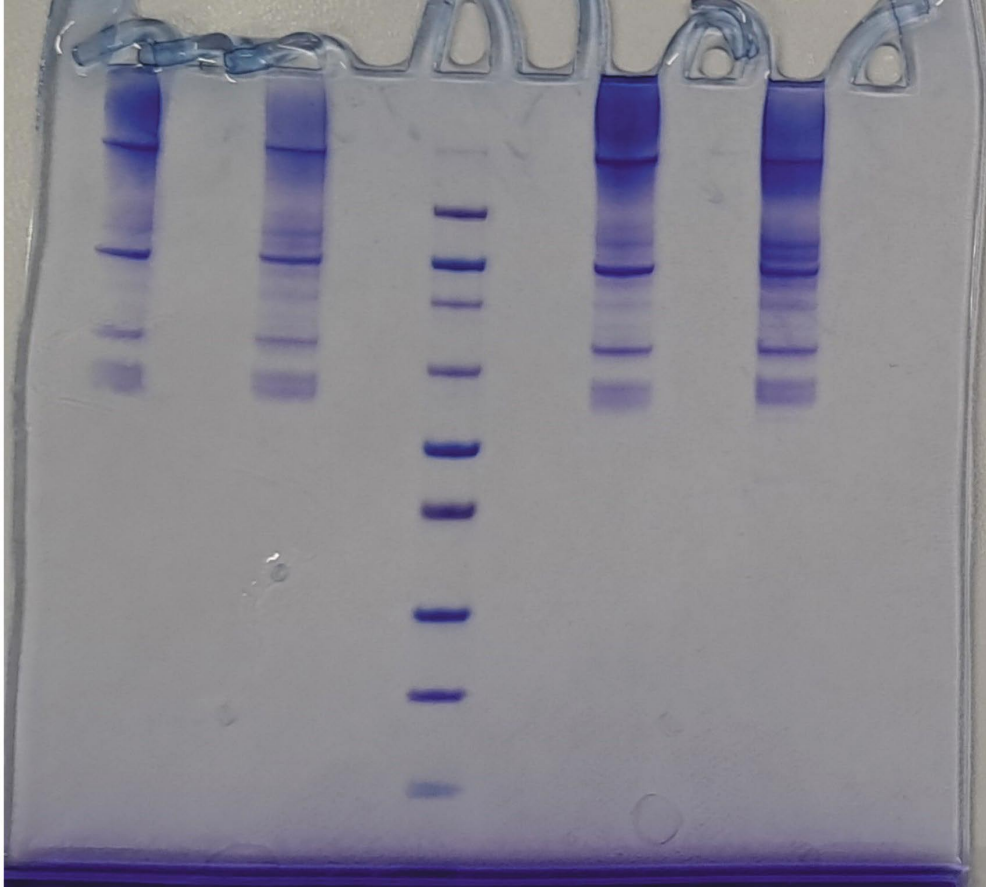

Supplement: SourceData FS4 — is the source file for Fig. S4. [file jgp_202513873_sourcedatafs4.pdf]
